# High-altitude hypoxia exacerbates dextran sulfate sodium (DSS)-induced colitis by upregulating Th1 and Th17 lymphocytes

**DOI:** 10.1080/21655979.2021.1975017

**Published:** 2021-10-19

**Authors:** Mohammed Gamah, Murad Alahdal, Yu Zhang, Yiling Zhou, Qiaorong Ji, Zhouyang Yuan, Ying Han, Xiangqun Shen, Yanming Ren, Wei Zhang

**Affiliations:** a Research Center for High Altitude Medicine, Key Laboratory for High Altitude Medicine, Ministry of Education, Qinghai University, Xining, Qinghai, 810001, China; b Medical College of Qinghai University, Xining, Qinghai, 810001, China; c Shenzhen Key Laboratory of Tissue Engineering, Shenzhen Laboratory of Digital Orthopedic Engineering, Shenzhen Second People’s Hospital (The First Hospital Affiliated to Shenzhen University, Health Science Center), Shenzhen P. R. China; d Medical Laboratory Department, Faculty of Medicine and Health Sciences, Hodeidah University, Al Hudaydah, Yemen; e Department of Pathophysiology, School of Basic Medical Sciences, Xuzhou Medical University, Xuzhou, Jiangsu, 221004, China; f Department of Pathology, West China Hospital, Sichuan University, Chengdu, Sichuan, 610041, China

**Keywords:** High altitude, hypoxia, dextran sulfate sodium, ulcerative colitis, inflammatory cells

## Abstract

High altitude hypoxia (HAH) involves the pathogenesis of ulcerative colitis (UC) and gastrointestinal erosions. However, the mechanism of effects of HAH in colitis remains controversial. This study reports the immunomodulation mediated by HAH to enhancing the severity of UC in the mice model. BALB/c mice were used to establish the UC model by dextran sulfate sodium (DSS) compared to wild type mice. Mice groups were exposed to hypoxic conditions in a hypobaric chamber with an altitude of 5000 m for 7 days. Then, Spleen, mesenteric lymph nodes and colon tissues were collected. The activity of UC, the infiltration of the immune cells, and the released cytokines were investigated. Results showed that the severity of DSS-induced UC significantly increased in mice exposed to HAH. The analysis of pathological changes showed increased weight loss and decreased colon length accompanied by diarrhea and bloody feces in the hypobaric hypoxia group. Interestingly, the levels of inflammatory cytokines IL-17, TNF-α, and IFN-γ in the spleen and mesenteric lymph node showed a significant increase within the colon of the hypobaric hypoxia group. The population of Th 1 and Th 17 cells in the spleen was significantly increased in mice exposed to hypobaric hypoxia compared NC group. Suggesting that high altitude hypoxia enhances colitis in mice through activating the increase of inflammatory Th1 and Th17 lymphocytes. In conclusion, this study revealed that hypobaric hypoxia directly increases the severity of UC in the mice model via increasing the activity of inflammatory CD4+ Th1 and Th 17 lymphocytes.

## Introduction

Ulcerative colitis (UC) is a category of inflammatory bowel disease (IBD) associated with weight loss, abdominal pain, bloody diarrhea, and intestinal barrier dysfunction [1,2]. These symptoms can become worse in patients who live in specific areas. Also, UC showed an association with some serious diseases including cardiovascular disease, tumor expansion, and emotional disorders such as depression and anxiety [3,4]. Though the etiology that promotes a pathogenesis development of UC is unclear, the environmental components are considered as intrinsic risk factors. Previous studies revealed that hypoxia appears to be a vital driver of inflammatory response leading to induce acute colon injury [5]. Several studies found that high-altitude disorders and hypoxia promote higher concentrations of inflammatory factors such C-reactive protein, interleukin-6 (IL-6), and interleukin-8 (IL-8) [6,7]. Further, recent studies revealed that animals exposed to hypobaric hypoxia at 4000 m above sea level showed an increase of intestinal barrier damage, inflammatory cell infiltrations, significant numbers of bacteria and poisonous chemicals invade the bloodstream, besides that reactive oxygen species are produced leading to oxygen local depletion [8–10]. Worthy mention, the disorder of intestinal barrier may induce various intestinal diseases and numerous organ dysfunction as a result of hypoxia [11]. Hence, the exposure to high altitudes could lead to gastric and duodenal erosion with consequent gastrointestinal bleeding [12–14]. Furthermore, the induced inflammation impairs intestinal barriers, which allows anoxia to expand formerly to normoxic tissue. A previous study conducted on 20 male suffering weight loss and intestinal disorders presented that hypobaric hypoxia plays a major role in the weight loss but the physiological mechanism remains unclear [15]. Another study focused on mountaineers revealed that many physiological diseases were associated with high altitude of hypoxia but the mechanism of internal diseases remains to be studied [16]. As known, in the intestinal epithelial cells, oxygenation increases at the base of the villi, while the tips of the villi are hypoxic [5]. In case of high altitude of hypoxia, hypoxia-inducible factor (HIF)-1α and HIF-2α are increased to maintain intestinal homeostasis. However, high concentration of HIF-1α and HIF-2α can promote inflammation and intestinal barrier injury. But a recent study reported that hypoxia may ameliorates inflammation of the colitis by modulating autophagy and mammalian target of rapamycin (mTOR)/NLRP3 pathway [17]. In contrast, another new study proved that treating hypobaric hypoxia reduced intestinal inflammation and improved a healing of mucosal barrier injury through inhibiting the immune responses [18]. Altogether, hypobaric hypoxia and high-altitude hypoxia could contribute in the regulation of intestinal dysfunction and colitis. However, the mechanism of effects of high-altitude hypoxia that enhance severe colitis development remains to be studied. Here, we aim to explore the pathological immunomodulation induced by high-altitude hypoxia that contributes to increasing colitis severity in the UC mice model.

## Material and method

### 2.1 Mice description and environment

Eight-week-old female BALB/c mice were purchase from Beijing Vital River Laboratory Animal Technology Co., Ltd. (Beijing, China). The mice were weighed and kept in plastic cages with woodchip bedstead in individually ventilated cages (IVC) and maintained below controlled environmental conditions at room temperature (22 ± 2◦C) with 50 ± 10% humidity and the switching cycle of 12 h light and 12 h dark. Mice allowed unlimited access to normal chow and tap water. They were maintained in a specific free pathogen facility at the Medical College of Qinghai University. Animal care was given under protocols endorsed by the Institutional Animal Care and Utilize Committee at the Medical College of Qinghai University.

### 2.2 Induction of UC and hypoxia treatment

Acute colitis was established by adding DSS (MW 36,000–50,000 Da, CAS no. 9011–18-1) to mice drinking water at 3.5% (wt/vol) ad libitum for 7 days [19]. BALB/c mice were indiscriminately categorized into four different experimental groups: Control group (NC), and DSS group (ND) to induce UC at 2270 m Xining city (36°37ʹ31.94”N, 101°45ʹ26.6”E); Hypobaric hypoxia group (HC), and hypobaric hypoxia DSS group (HD) were exposed to a simulated altitude of 5000 meters (atmospheric pressure is 405 mmHg) in decompressing chamber for seven days (HCP-III, laboratory animal low-pressure simulator, 1.4 m(long) × 0.8 m(diameter), frequency of ventilation 10 times/h. Shaanxi Science and technology resources center, Xi’an, China) to emulation hypoxia [20]. The food and water allocate to the HC and HD groups were the same as those for the NC and ND groups. The temperature was 22 ± 2°C, and the light/dark period was 12 h. Mice were sacrificed through cervical spinal dislocation on the day 8 for a collection of spleen, mesenteric lymph nodes, and colon tissue for more analysis.

### 2.3 Disease activity index (DAI)

To assess the extent of colitis. Mice were tracked daily for monitoring body weight loss, stool consistency, visible blood, and rectal bleeding. Disease activity index (DAI) has been measured according to Whittem et al. [21]. Scores were evaluated as follows: stool consistency was scored as standard (0), soft (1), very soft (2) and, diarrhea (3); the rectal bleeding was scored as (0) no blood; (1) red; (2) dark red and (3) gross blooding; visible blood scored as (0) standard, (1) red, (2) dark red and 3 black resulting in the total DAI a score ranging from 0 to 9.

### 2.4 Samples collection

Colonic samples (*n* = 5/group) were assembled from BALB/c female mice and photographed. The collection was conducted on days 8. Then, the colon length has been calculated and reported as an indirect inflammation indicator. The colon was then gently cleared of feces. The colon tissue has been cut into three parts (proximal, middle, and distal). The distal section was used for histology. Middle and proximal portions were frozen in liquid nitrogen for the quantification of myeloperoxidase (MPO) activity and qRT-PCR. The entire spleen and mesenteric lymph nodes (MLNs) were collected. They were mechanically dissociated and lysed with red blood cells lysis buffer. Single-cell suspensions were passed through nylon membranes and centrifugated at 475 x g for 5 min at 4°C. The precipitation was re-suspended with cDMEM (Gibco, Thermo Fisher Scientific, China). Then it was used for flow cytometry and ELISA.

### 2.5 Histopathological examinations

At necropsy, colon tissue was assembled, fixed in OCT compound, and then saved at −80°C. Five-micrometer (5 μm) sections were cut on a LEICA CM1950 Cryostat (Leica Biosystems) and fixed in ice-cold 4% paraformaldehyde then stained with hematoxylin and eosin for histological evaluation. Histological scoring was based on two parameters as indicated in Table 1. The histological score was calculated by adding the two evaluations and giving a maximal score of 8. The tissues were assessed under a light microscope.

**Table 1.** Scores of intestinal tract inflammations

Severity	Score 1	Extent	Score 2
No inflammation	0	No inflammation	0
Mild 10–25%	2	Mucosa	1
Moderate 26–50%	3	Mucosa and submucosa	2
Marked >51%	5	Transmural	3

### 2.6 Measurement of Myeloperoxidase Activity

Briefly, colon tissue was weighted, then the suitable amount of medium for a 5% homogenate was added, and smashing until it was fully homogenized. The other steps were carried out according to the guidance of MPO detection kit (Abcam, USA), the absorbance value was measured with a micro reader at 460 nm. The results were calculated according to the formula (U/g) of wet tissue weight.

### 2.7 Assay of cytokine

To measure cytokine production 5 × 10^6^ CD4 + T cells from spleen and MLN were seeded in 1 ml of cDMEM (Gibco, Thermo Fisher Scientific, China) Complete Medium contains high glucose medium with 2 mM L-glut, sodium pyruvate, and 10% FBS, termed complete cDMEM, in the presence of 0.5 mg/ml plate-bound anti-CD3ε (145–2C11) monoclonal antibody on flat-bottom 48-well plates (Costar) at 37°C in a humidified environment incubator containing 5% CO_2_ for 72 h. Culture supernatants were obtained and the production of cytokines such as IFN-γ, IL-17, TNF-α, and IL-10 was investigated. The protein concentrations were ascertained using specific Enzyme-Linked Immunosorbent Assay (ELISA) kits (BPS Bioscience, USA) according to the producer instructions, including IFN-γ, TNF-α, IL-10, and IL-17 were applied to measure cytokine levels.

### 2.8 Flow cytometry assay

For the intracellular staining of IL-17 and IFN-γ. The splenic mononuclear cells were stimulated in vitro for 4 h with 2 μg/mL of phorbol 12-myristate 13-acetate (PMA) and 1 μg/mL of monensin. Then, it was tested by flow cytometry as stated in Alahdal et al. [22]. Briefly, cells were washed and stained with phycoerythrin (PE)-conjugated anti- CD4 antibodies, the cells were fixed overnight with 4% paraformaldehyde (Fisher scientific, USA). Cells were permeabilized and stained for 30 min at 4°C with FITC-conjugated anti-IL-17, INF-γ antibodies (Bio Legend, USA). The data were collected with CYTOFLEX (BECKMAN COULTER) and analyzed by Cytexpert software. Cells were analyzed by setting gates on FSC-A scatter according to unstained control compared to positive control of every antibody.

### 2.9 Quantitative Real-time PCR (qRT-PCR) analysis

On day 8, colons were detached and homogenized to extract total RNA with TRIzol reagent (Invitrogen™, USA) according to the constructer’s instructions. The RNA concentration and purity were determined by measuring the absorbance at 260 and 280 nm. Complementary DNA (cDNA) was reverse transcribed from 2 μg RNA using the cDNA synthesis kit (Takara, China). qRT-PCR was carried out using TB Green Premix Ex Taq ll in a CFX96 Real-Time Detection System (Bio-Rad, Hercules, CA, USA). Relative levels of target mRNA were compared with GAPDH using the 2^−∆∆Ct^ method. The sequences of used primers are listed in Table 2.

**Table 2.** Primers used in this study

Primer	Orientation	Sequence (5ʹ–3ʹ)
GAPDH	F	TGGAATCCTGTGGCATCCATGAAAC
	R	TAAAACGCAGCTAGTAACAGTCCG
IL-17	F	CCACGTCACCCTGGACTCTC
	R	CTCCGCATTGACACAGCG
IFN-γ	F	TCAAGTGGCATAGATGTGGAAGAA
	R	TGGCTCTGCAGGATTTTCATG
TNF-α	F	CCCTCACACTCAGATCATCTTCT
	R	GCTACGACGTGGGCTACAG

### 2.10 Statistical analysis

The data are reported as means ± SD. Mice number used refers to *N*. The statistical differences between groups were determined by two-way ANOVA followed by Bonferroni post-hoc test, or one-way ANOVA followed by Tukey post-hoc test. Statistical analyses were carried out with GraphPad Prism®5 software (GraphPad Software Inc., San Diego, CA). A *P*-values of less than 0.05 (*P* < 0.05) were considered to be statistically significant.

## Results

UC is one of the common gastrointestinal diseases leading to intestinal dysfunction. Many intrinsic risk factors involve the development of UC including biological and environmental factors. Hypoxia inducible factors were described early as vital inducers of intestinal inflammatory injury that elevate colitis development. However, few studies reported the effect of high-altitude hypoxia effects on the colitis. This study reports that hypobaric hypoxia significantly contributes to enhance the severity of UC through activating Th1 and Th-17 leading to increase inflammatory responses.

### 3.1 Effect of high-altitude hypoxia on in UC induced mice

**Figure 1.**
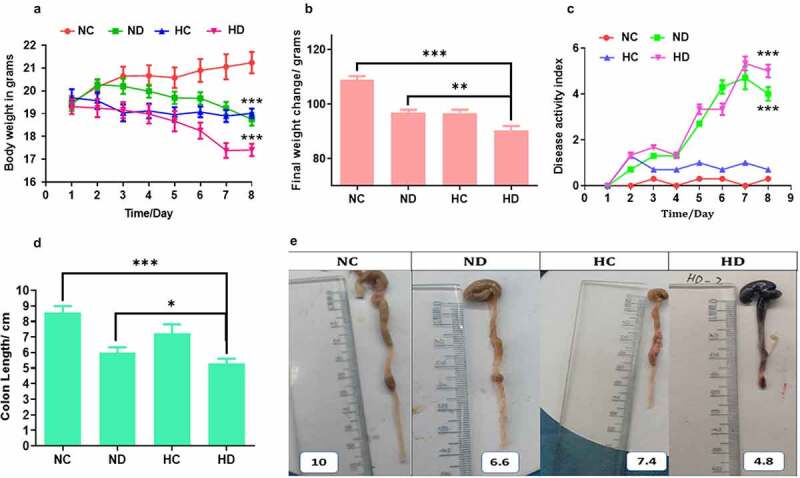
High-altitude hypoxia enhances the severity of ulcerative colitis in the DSS induced colitis mice model, 5 mice /group were used. (a) The effect of high-altitude hypoxia on the body weight during 8 days among groups (NC, normal group; ND, DSS group; HC, Hypobaric hypoxia group; HD, hypobaric hypoxia DSS group). (b) The effect of high-altitude hypoxia during 3 months among tested groups. (c) the results of disease active index among tested groups. (d, e) The length of colon among tested groups. *P < 0.05, **P < 0.01, ***P < 0.001

In order to test the effect of HAH on the immunomodulation during induced colitis, DSS colitis model was established. The observation of symptomatic indicators after 4 days obviously exhibited reduced mobility, body weight loss, diarrhea, and rectal blood. Importantly, the weight loss was significantly noticed severe in the mice of HD group compared to NC and ND groups (*P* < 0.001, and *P* < 0.05) respectively, especially on days 6, 7, and 8 as presented in Figure 1 (A, B). Further, DAI showed significant high scores in the HD group when compared to NC and HC groups, especially in the final three days as shown in Figure 1(C). In addition, rectal bleeding was obviously observed in the HD group more than those in the ND group. The measuring of colon length showed that mice in HD group were significantly shorter than that of the NC and ND groups on day 8 (*P* < 0.001 and *P* < 0.05) respectively, see Figure 1 (D, E). Further, mice in the HC group exhibited no significant differences compared to NC group. Collectively, these results proved that HAH aggravates clinical severity of DSS-induced colitis.

*3.2 High-altitude hypoxia enhances intestinal epithelial damage in UC induced mice through increased neutrophil infiltration*

**Figure 2.**
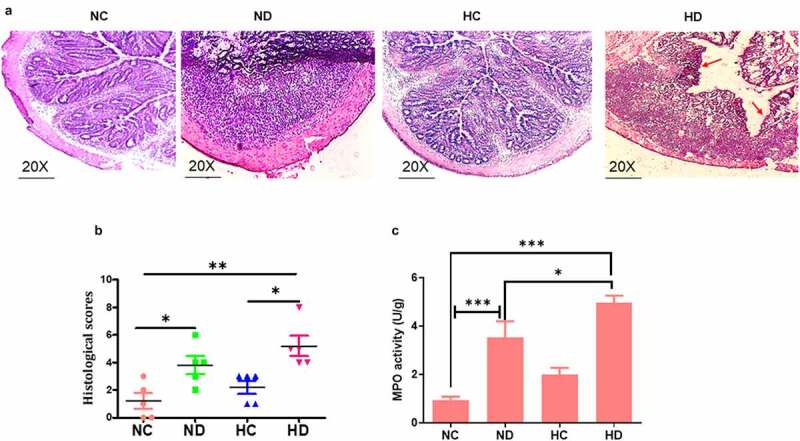
HAH showed significant damage of colon epithelial layer and activation of myeloperoxidase (MPO) in the DSS induced colitis model, 5 mice /group were used to test: (a) HE histology presented the damage of epithelial layer as pointed by red arrow; (b) the score of histological inflammation of colon among animal tested groups; (c) MPO activity scores among tested groups. *P < 0.05, **P < 0.01 ***P < 0.001

Sections of colon tissue in the HD group compared to NC groups exhibit critical damage of epithelial architecture and severe inflammatory cell infiltration with the extent to mucosa and submucosa layer as seen in Figure 2 (A, B). The analysis of MPO activity showed significant increase of MPO in the HD and ND groups compared to NC group (*P* < 0.001) as shown in Figure 2 (C).

### 3.3 High-altitude hypoxia increases inflammatory cytokines in UC induced mice

**Figure 3.**
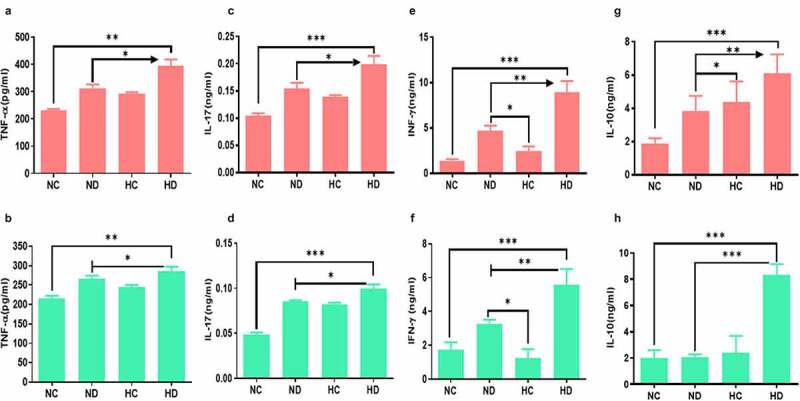
Effect of high-altitude hypoxia on the spleen and MLN cytokines. Splenocytes and MLN were detached freshly (5 × 10^6^) from BALB/c mice (5 mice /group) and cultivated in the presence of anti-CD3+ antibodies for 72 h, and then ELISA was used to measure IL-17, IFN-γ, IL-10, and TNF-α levels. (a, b) The levels of TNF-α in the spleen and MLNs respectively. (c, d) The levels of IL-17 in the spleen and MLNs respectively. (e, f) The levels of IFN-γ in the spleen and MLNs respectively. (g, h) the levels of IL-10 in the spleen and MLNs respectively. *P < 0.05, **P < 0.01, ***P < 0.001

**Figure 4.**
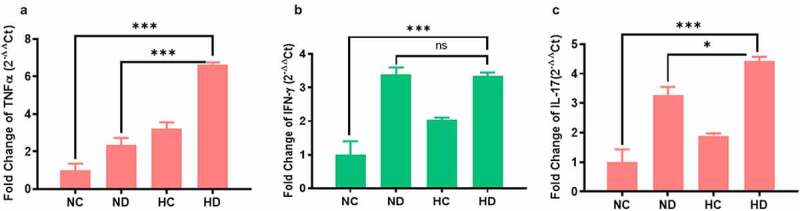
The gene expression changes of inflammatory cytokines in the colon tissue collected from four tested groups (5 mice /group) showed increased inflammatory cytokines in the colon tissue. (a) The expression changes of TNF-α in the four tested groups. (b) The expression changes of INF-γ in the four tested groups. (c) The expression changes of IL-17 in the four tested groups. **P < 0.01 ***P < 0.001

In order to explore the inflammatory responses under the effect of HAH in UC induced mice, levels of IL-17, IL-10, TNF-α, and INF-γ cytokines were tested by ELISA. The levels of TNF-α have significantly increased in the spleen of both HC and HD groups compared to ND group (*P* < 0.001), see Figure 3 (A). In addition, the levels of TNF-α in MLNs were also significantly higher in HC and HD groups compared to NC group (*P* < 0.001) as presented in Figure 3 (B). Levels of IL-17 in spleen and MLNs were also significantly higher in HD group compared to NC group (*P* < 0.001), see Figure 3 (C, D). Moreover, the levels of IFN-γ were found higher in HD group both in spleen and MLNs compared to NC and HC groups as presented in Figure 3 (E, F), as well as the IL-10 was significantly elevated in the spleen and MLNs of HD group compared to NC group (*P* < 0.05; *P* < 0.001) respectively as shown in Figure 3 (G, H). These findings confirmed by analyzing the expression of inflammatory cytokines in the colon tissue using qPCR. Results as seen in Figure 4 (A, B, C) showed significant increase of inflammatory cytokines in colon of HD group compared to NC group TNF-α (*P* < 0.001), INF-γ (*P* < 0.01), IL-17 (*P* < 0.001).

*3.4 High-altitude hypoxia mediates immunomodulation through increasing CD4^+^IL-17^+^ lymphocytes and CD4^+^INF-γ^+^*

**Figure 5.**
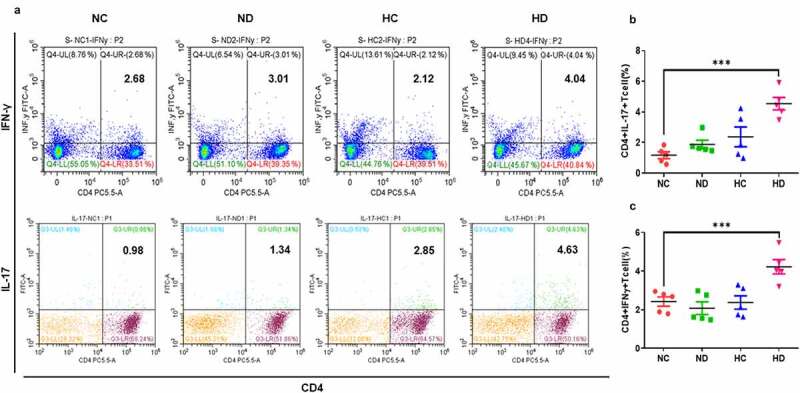
HAH significantly increases the percentage of infiltrating CD4+ IFN-γ+ cells and CD4+ IL-17+ cells in the DSS induces colitis groups compared to other tested groups, 5 mice /group were used in this analysis. (a) The percentage of CD4+ IFN-γ+ and CD4+ IL-17+ lymphocytes in the spleen and MLNs samples of all tested groups. (b) The population of CD4+ IFN-γ+ in the all tested groups. (c) the population of CD4+ IL-17+ in the all tested groups

The analyzing of lymphocytes in the spleen and MLNs that were collected from UC mice model presented that CD4^+^ IL-17^+^ and CD4^+^ IFN-γ^+^ Th1 cells significantly increased in the spleen of HD group compared to NC group. Suggesting that HAH promotes the proliferation of inflammatory T helper cells in lymphoid organs as seen in Figure 5 (A, B, C) leading to enhancing the severity of UC in mice model.

## Discussion

UC is a common intestinal inflammatory disease. The precise etiology of UC still unknown and superior therapy remains to be reported [23]. However, the severity of UC can be promoted by several biological and environmental factors [24]. Some scientists believed that environmental and immunological changes could play a role in the development of UC [25]. Recently, the relation between hypoxia and intestinal inflammatory diseases including colitis was reported. Hypoxia resistance can increase the inflammatory responses in the intestinal tract thereby inducing UC and other serious diseases [26]. In this study, BALB/c mice were enrolled to establish UC animal model and investigating the effect of high-altitude hypoxia on the colitis development and to explore the mechanism of effect. Hypoxia chamber was used for a high altitude of 5000 m according to previous study [27]. Results showed that HAH significantly increases the severity of UC, which suggest a warning for those people suffering IBD disorders or colitis to avoid living in high areas and be careful of long flight trips. Our outcomes presented notably aggravated clinical symptoms of UC through diminished bodyweight and colon portion conducted with the occurrence of diarrhea, blood in feces, and inflammatory cell infiltration, thereby boosting the disease activity index scores which are in line with the preceding studies [28,29]. Myeloperoxidase (MPO) is a proinflammatory marker observed within neutrophils and less in macrophages, and this enzyme intensifies host protection against bacteria [30]. The increased activity of MPO in both the ND and HD groups in animal model suggesting colonic infiltration of neutrophils that was induced under the effect of HAH. Further, the increased inflammatory cytokines such as TNF-α and IFN-γ under the effect of HAH expanded the permeability of tight junction and induced apoptosis of intestinal epithelial cells and subsequently enhance the severity of UC [20,31]. Furthermore, an imbalance in pro-inflammatory and anti-inflammatory cytokines is a significant cause of intestinal mucosal injury [32]. Indeed, previous findings have been observed a robust association between abnormal intestinal permeability and mucosal inflammation in UC or volunteers and laboratory animals exposed to hypobaric hypoxia [33,34]. Here, we analyzed the most respond immune cells for HAH induced UC in animal model. We noticed that Th1 secreting IFN-γ and IL-17 producing lymphocytes particularly IL-17 + T cells have been activated significantly in the HD group. These results support a previous study concluded that HIF-1α is required for the TH17 function [35,36]. Hence, the association between the seriousness of UC and increased levels of IL-17 has been clearly identified. The activation of Th17 corresponds with the parallel induction of Treg is a feature of resistance to colitis disease [37]. What is unusual, we noticed the increase of IL-10 levels that associated with the increase of UC severity. Although IL-10 is a regular immunosuppressive cytokine, there are deviations of IL-10 concentrations in IBD. The intestinal IL-10 expression levels were either the same or higher in IBD patients than in healthy controls [38]. Hence, hypoxia might work to convert CD4 cells to Treg cells to inducing immune resistance, which might be one of the reasons that Treg increases in hypoxia induced colitis [39]. Therefore, our study is the first study to explore the activated T lymphocyte subtype in response to high altitude hypoxia in the DSS mice model.

## Conclusion

This study demonstrates the mechanism of effect of HAH on the intestinal immune populations leading to enhance the severity of DSS-induced colitis in mice. Collectively, our data revealed that high altitudes hypoxia boosts the severity of colitis in the DSS murine model by increasing IFN-γ and IL-17 cytokines in accompany to activation of Th1, and Th17 lymphocytes as presented in the graphical abstract.

## Data Availability

Authors declare that all data belong to this manuscript have been included in the manuscript more details are available by sending email to the corresponding author. https://www.jianguoyun.com/p/DYhkB-8Q1_zuCRiSzJAE; https://www.jianguoyun.com/p/DYBfWIMQ1_zuCRiUzJAE; https://www.jianguoyun.com/p/DeYcqxcQ1_zuCRiMzJAE

## Abbreviations

UC: ulcerative colitis, DSS: dextran sulfate sodium, ELISA: enzyme-linked immunosorbent assay, qRT-PCR: quantitative real-time PCR, DAI: disease activity index, IBDs: inflammatory bowel disease.
